# A Randomised Control Trial and Comparative Analysis of Multi-Dimensional Learning Tools in Anatomy

**DOI:** 10.1038/s41598-020-62855-6

**Published:** 2020-04-09

**Authors:** Chris Wang, Ben Kei Daniel, Mustafa Asil, Prashanna Khwaounjoo, Yusuf Ozgur Cakmak

**Affiliations:** 1 Department of Anatomy, School of Biomedical Sciences, University of Otago, Dunedin, New Zealand; 2 Education Technology Group, Higher Education Development Centre, University of Otago, Dunedin, New Zealand; 3 Centre for Healthcare Education and Research Innovation (CHERI), School of Medicine, Medical Sciences and Nutrition, University of Aberdeen, Aberdeen, United Kingdom; 4 Brain Health Research Centre, Dunedin, New Zealand; 5 Medical Technologies Centre of Research Excellence, Auckland, New Zealand; 6 Centre for Health Systems and Technology, University of Otago, Dunedin, New Zealand

**Keywords:** Anatomy, Medical research

## Abstract

This article presents the results of a study that examined students’ ability to retain what they have learned in an anatomy course after thirty days via using various learning tools for twenty minutes. Fifty-two second-year medical students were randomly assigned to three learning tools: text-only, three-dimension visualisation in a two-dimensional screen (3DM), or mixed reality (MR). An anatomy test lasting for twenty minutes measuring spatial and nominal knowledge was taken immediately after the learning intervention and another thirty days later. Psychometric tests were also used to measure participants’ memory, reasoning and concentration abilities. Additionally, electroencephalogram data was captured to measure the participants’ awakeness during the learning session. Results of this study showed that the MR group performed poorly in the nominal questions compared to the other groups; however, the MR group demonstrated higher retention in both the nominal and spatial type information for at least a month compared to the other groups. Furthermore, participants in the 3DM and MR groups reported increased engagement. The results of this study suggest that three-dimensional visualiser tools are likely to enhance learning in anatomy education. However, the study itself has several limitations; some include limited sample size and various threats to internal validity.

## Introduction

Learning methods have been continually changing over time with the increasing employment of learning technology^1–3^. The ease and access of mobile platforms and the internet have provided new methods of communication and teaching. Following this trend, the use of three-dimension (3D) tools in classrooms and universities has been explored and has provided new possibilities for teaching^1–4^. A particular area this type of learning has proved useful is for anatomy where 3D learning tools can enhance the understanding of the spatial associations within the human body, since anatomical structures in the body interact with each other in three-dimensional space^5^. The use of cadaver-dissections in learning human anatomy is viewed by many anatomists as the gold-standard in anatomy teaching^6,7^. However, researchers have identified a number of challenges associated with dependency on the use of cadavers, some of these include the expense of time, money, and expertise in preparing, maintaining, storing/disposal/access of cadavers; cadaver variability vs alive humans; and finally the potential health hazards associated with cadaver dissections^1,8^. In addition to these challenges, there is a noticeable decline in the allocation of anatomy teaching hours in New Zealand and Australia^9^, necessitating experimenting with innovative ways of teaching anatomy such as the use of 3D visualizers^1^.

Currently, there is a growing body of literature on the efficacy of three-dimensional visualisation on a two-dimensional screen (3DM) learning tools compared against conventional teaching (e.g. lecture and textbook-style learning tools)^10–12^. 3DM refers to the ability to display a 3D object on a two dimensional (2D) surface^13^. The literature has mixed views on the benefits associated with using 3DM to learn nominal information (names of structures)^14,15^. However, spatial information acquisition is generally considered positive when using 3DM learning tools^12,16^. Research has also reported that students prefer lectures that are supplemented with 3D models^17^. It was also reported elsewhere that the use of 3DM has been rated by students to be of equal effectiveness^18^ or more helpful than 2D  computed tomography images^15^.

Similar to 3DM, mixed reality (MR) is a type of 3D visualizer, which can use a head-mounted augmented reality device. MR involves deploying virtual objects into the real environment^19,20^. For instance, using holograms shown by a head-mount display. The ability of MR to afford significant improvements in factual knowledge is well supported in the literature^21,22^. The acquisition of nominal information compared to the use of textbooks^22^ is also considered to be superior. Furthermore, students who use MR tend to demonstrate significantly higher accuracy in solving spatial questions when compared to 2D instructions^23^. There are also benefits of reduced cognitive load using MR when compared to 3DM learning tools^4,24,25^ and students have significantly preferred the use of MR compared with 3DM^26–28^ and textbook-style learning tools^4^. Overall students using MR learning tools reported increased motivation^29,30^, enjoyment^26,30^, immersion^26,27,29,31^, 3D model comprehension^29^, safety^32^, confidence^26^, exploratory behaviour^28^, creativity^27^, and concentration^26^.

A number of studies have investigated the use of MR in teaching, however to the best of our knowledge, research into how the various techniques mentioned above support long-term retention of information is lacking. Herein we present our study exploring the ability of MR in supporting long-term retention outcomes, as well as exploring the short-term knowledge retention. The study also qualitatively explored the differences of experiences using MR compared to 3DM and textbook-style learning tools.

## Methods and sample

All second-year University of Otago medical students were invited to participate in this project. The incentive for participating were the benefits of learning and participating to interact with the MR and a guided multi-user learning experience of the visual pathway (part of their third-year curriculum). No monetary incentive was given.

The inclusion criteria for the study required participants to be studying second-year medicine at the University of Otago, New Zealand. The exclusion criteria were as follows: (a) those who had a medical history of motion sickness or vertigo; (b) a medical history of a seizure or epilepsy; (c) are prescribed long-term use of medication.

The study sample included 52 second-year University of Otago medical students (34 males, 18 females) between 19 and 31 years of age. They were randomly distributed into three treatment groups: text-only group (*n* = 18), 3DM group (*n* = 15), MR group (*n* = 19).

All participants were given the opportunity to read through the participation information sheet and consent form. A signed consent form was also obtained. In both the information sheet and consent form, participants were informed they were able to withdrawal at any moment without consequences. This project was reviewed and approved by the University of Otago Human Ethics Committee, New Zealand (reference: H18/074). This research was performed in accordance with the Declaration of Helsinki Ethical Principles for Medical Research involving Human Subjects. Note, one participant was lost during follow up.

## Experiment procedure

**Figure 1:**
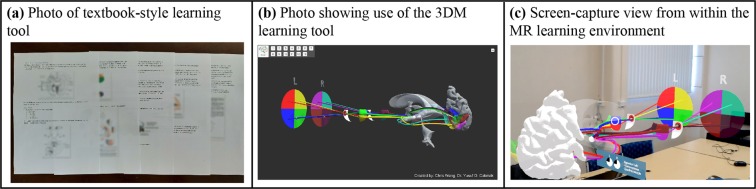
Pictures of different learning tools.

The participants were randomly split into 3 different groups. Participants in the text-only group received a textbook-style learning tool which consisted of text and 2D images from two clinical neuroanatomy textbooks (Fig. 1a): Clinical Neuroanatomy and Neuroscience, sixth edition, authored by Estomih Mtui, Gregory Gruener, M. J. T. FitzGerald^33^; Basic Clinical Neuroanatomy, first edition, authored by Paul A. Young, Paul H. Young^34^. The 3DM group were provided with the same text with the addition of a laptop and mouse. On the laptop, the 3D visualizer software of the visual pathways was preloaded and had the controls of rotation, panning, selecting different models and a reset button (Fig. 1b). The MR group received the same text with the addition of the Microsoft HoloLens device with the preloaded 3D hologram visualizer software (Fig. 1c). The models loaded in the HoloLens were the same as the 3DM laptop program. The controls of the HoloLens included loading, reloading, or deleting 3D models from the environment.

The experiment was split into two sessions one month apart. The participants were also requested to not complete any study related with the visual pathway system between session 1 and session 2. Session 1 and session 2 were completed over the course of a week. In session 1, the participants completed tasks in the following order: (a) demographics and academic background survey, (b) orientation of learning tool, (c) learning session, (d) anatomy test 1, and (e) user perception and usability survey. The tasks for session 2 were in the order as follows: (a) anatomy test 2, (b) Cambridge Brain Sciences’ (CBS) psychometric tests, (c) multi-user hologram experience,and (d) memorability and long-term retention survey. It should be noted that all participants will complete the multi-user hologram experience so none of them are disadvantaged from the learning material.

The learning session in session 1 was limited to twenty-minutes. During the learning session, the participants’ electroencephalogram (EEG) was recorded providing a minute-by-minute bispectral index (BIS). The BIS number is a quantitative measure of awakeness. The participants’ minute-by-minute learning actions were also recorded as one of three activities: (a) using only the text, (b) using only the device, and (c) using both text and device.

## Instruments

### Demographics and academic abilities survey

This survey obtained information about participants’ sex, ethnicity, age, pathway of entry into medicine, previous academic experience, and previous anatomy paper grades (see supplementary file appendix 1). The purpose of this was to understand the demographics and academic background of the students. This survey was completed before the beginning of the experiment.

### Bilateral electroencephalogram (EEG) recording

An FDA approved BIS monitoring system (BIS Complete 4-Channel Monitor and a BIS LoC 4 Channel OEM Module) to record and interpret the minute-by-minute EEG signals of the participants during the learning process.

### Anatomy test

Anatomy test 1 and anatomy test 2 consisted of the same question items but arranged differently in a random order (see supplementary file appendix 2). The test was developed under the supervision of the primary investigator with over 18 years of anatomy teaching experience. The format of answering each question item was an open short-answer response. A marking rubric was also developed to ensure consistent and reliable  marking (see supplementary file appendix 3). One point was allocated to each correct response. The tests had three types of questions, with 6 question items of each type: (a) nominal type questions, (b) spatial type questions, and (c) both nominal and spatial (mixed) type questions. Nominal questions only required participants to provide the name of structures and spatial questions consisted of the relationship between given structures. Finally, in mixed type questions, participants were required to recall both the naming and relationship between structures to solve the question.

### User perception and usability survey

User perception and usability survey consisted of eleven 5-point Likert-scale questions which allowed the students to rate the learning tool on various aspects such as engagement, ease of use, and confidence (see supplementary file appendix 4). Two of these questions on engagement allowed students to explain with an open response why they provided a specific rating.

### Memorability and long-term retention survey

Memorability and long-term retention survey consisted of three 5-point Likert-scale questions which focused on confidence, memorability of the learning session, and self-perception of the effectiveness of the learning session (see supplementary file appendix 5). All three questions allowed students to explain their rating with an open response.

### Cambridge Brain Sciences’ (CBS) psychometric tests

Cambridge Brain Sciences’ (CBS) psychometric tests have been employed to research divisions of human intelligence^34^. The psychometric tests were completed on the same laptop and mouse which ran the 3D visualizer program through an Online Cognitive Assessment Platform created by CBS. Eight psychometric tests were used to examine the participants’ memory (spatial short-term memory, visual spatial working memory, and episodic memory); reasoning ability (mental rotation, visuospatial processing, and deductive reasoning); and concentration (response inhibition and attention).

## Data Analysis and statistics

Quantitative and qualitative analyses were used to investigate the effectiveness of learning spatial and nominal anatomy knowledge and the usability of the learning tools: text-only, 3DM, and MR. The quantitative data were analyzed using IBM Statistical Package for Social Sciences (SPSS) 25.0 and Microsoft Excel 2016. For qualitative data, the open answers from the questionnaires were compiled into key identified themes. Then a thematic analysis was performed using NVivo 11 and the themes of participant responses were explored through word clouds. The exemplars of identified themes and associated quotations are provided in supplementary file appendix 6.

An alpha value (α) of 0.05 (level of significance) was used for each analysis. We reported effect size measures (partial eta-squared) to indicate the magnitude of the effect of interest. Partial eta squared measures in the quantitative data were defined as small (η_p_^2^ = 0.0099), medium (η_p_^2^ = 0.0588) and large (η_p_^2^ = 0.1379)^35^.

The data analysis was broken down into three sections to compare the effectiveness of the learning tools in the following: (a) anatomy knowledge acquisition; (b) long-term anatomy retention; (c) usability and user perception of learning tools.

Firstly, we investigated if there were any benefits or disadvantages in using 3D visualizers as an adjuvant to textbook materials on influencing knowledge acquisition rates of different anatomical knowledge aspects. We employed Analysis of Variance (ANOVA) with Bonferroni post-hoc analyses to test whether treatment groups differed in their achievement on nominal, spatial and mixed sections of the anatomy test 1. The anatomy test 1 section scores were correlated with the CBS subscale scores to explore the relationships between short-term retention and cognitive abilities within each group.

Secondly, we examined if there were any benefits in using 3D visualizers as an adjuvant to textbook materials on influencing long-term memory retention rates of different anatomical knowledge aspects. We employed Exploratory Factor Analysis (EFA) to understand the underlying factor structure of the questionnaire items used. We selected the factors if their eigenvalue was >1 and the number of factors was determined using the scree test results, as well as the interpretability of the factors. A mixed ANOVA with Bonferroni post-hoc analysis was employed to compare each group on the achievement differences of anatomy test 1 and anatomy test 2 on nominal, spatial and mixed sections.

Lastly, we explored the differences in the usability and perception of the learning tools (textbook-style, 3DM, and MR). The item responses of the user perception and usability survey and memorability and long-term retention survey were compared between the groups. The qualitative feedback was also explored, identifying key themes. A one-way ANOVA was also performed to compare the mean EEG BIS data between groups to identify potential differences in the awakeness level of the participants during the learning session.

## Results

### Knowledge acquisition

The baseline anatomy academic abilities can be found in supplementary file appendix 7. A one-way ANOVA showed significant differences for only nominal type questions in anatomy test 1 scores when comparing the learning groups, *F*(2,49) = 13.722, *p* < 0.001. Bonferroni post-hoc analyses indicated that the average number of correct answers was significantly lower in the MR group (*M* = 2.32, *SD* = 1.81) compared to the text-only group (*M* = 4.83, *SD* = 1.20, *p* < 0.001) and 3DM group (*M* = 3.93, *SD* = 1.62, *p* = 0.008). There were no significant differences in the performance of the learning groups when comparing spatial type questions (*F*(2,49) = 0.353, *p* = 0.704) or mixed type questions (*F*(2,49) = 0.776, *p* = 0.466).

Pearson correlations were calculated to examine the relationship between students’ anatomy test 1 scores and psychometric test performances within each treatment group. A significant positive correlation was found within the text-only group between students’ nominal type questions scores and their attention scores (*r* = 0.572, *p* = 0.016). Another significant positive correlation was identified within the MR group between students’ spatial type questions scores and their spatial short-term memory scores (*r* = 0.609, *p* = 0.006).

### Long-term knowledge retention

**Figure 2:**
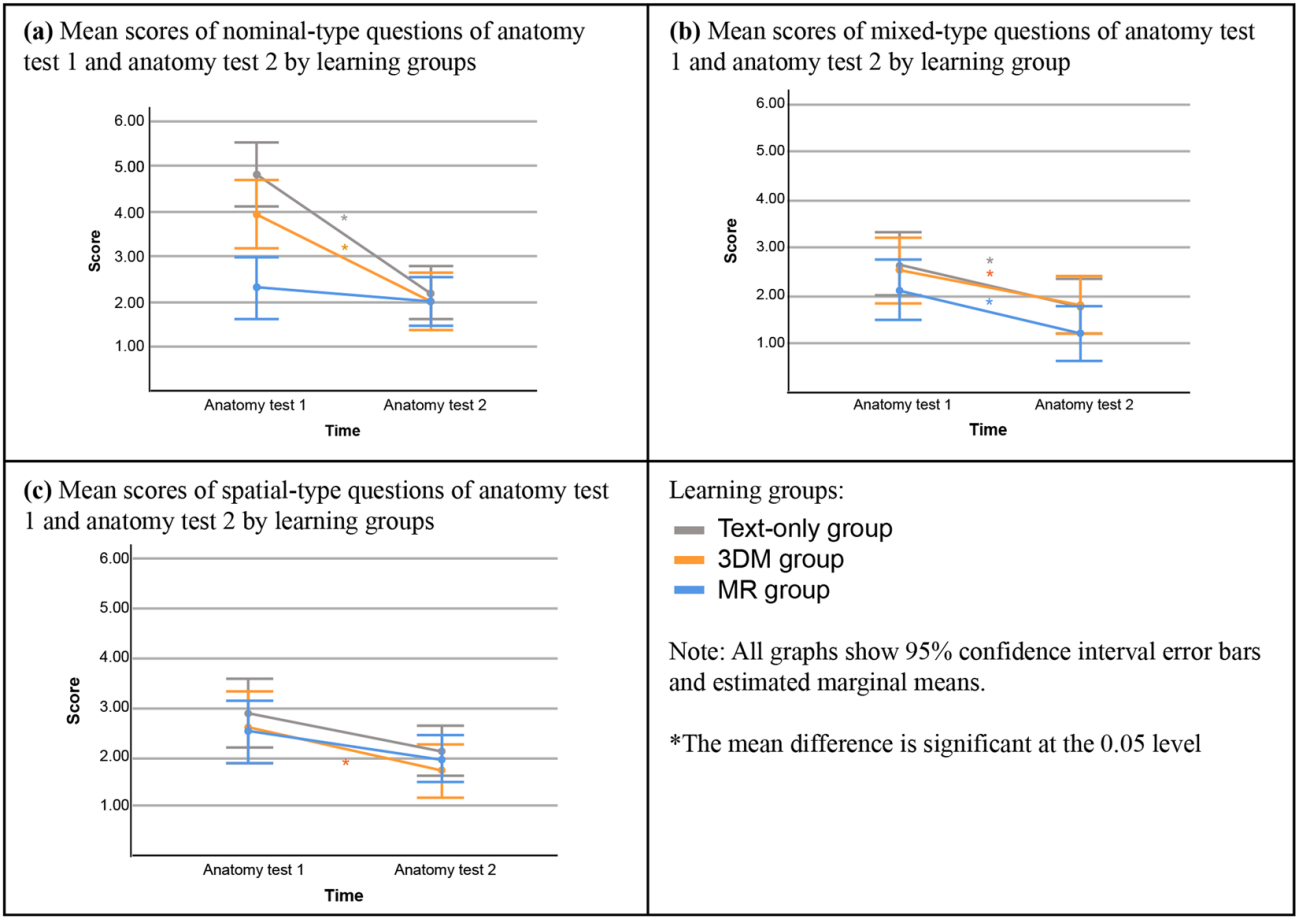
Comparing anatomy test 1 performance with anatomy test 2 performance within each group by question type.

A mixed ANOVA showed significant differences when comparing the learning groups’ change in question type scores over time (*F*(2,48) = 4.787, *p* = 0.013). In a more detailed analysis, there were also indications of significant differences when comparing the differences in anatomy test scores between the learning groups over time (*F*(2,48) = 5.624, *p* = 0.006) and question types performance between learning groups (*F*(2,48) = 3.566, *p* = 0.036). Bonferroni post-hoc analysis was performed to examine the changes over time within each group on each question type. The findings are summarized in Fig. 2.

When comparing anatomy test 1 and anatomy test 2 within the text-only group (shown in Fig. 2a), there was a significant decrease in nominal type question score (2.65 points, η_p_^2^ = 0.58, Power = 1.00, p < 0.001) and mixed type question score (0.88 points, η_p_^2^ = 0.17, Power = 0.87, p = 0.003). However, when comparing spatial type question means, there was a non-significant decrease of 0.77 points (η_p_^2^ = 0.08, Power = 0.49, p = 0.053). This means that spatial type information seems to be retained, but nominal and mixed type questions were not retained.

Comparing the 3DM group anatomy test 1 question scores to anatomy test 2 question scores, they had a significant decrease in all three question types (Fig. 2b); nominal question type (1.93 points, η_p_^2^ = 0.39, Power = 1.00, p < 0.001), mixed question type (0.73 points, η_p_^2^ = 0.11, Power = 0.67, p = 0.018), spatial question type (0.87 points, η_p_^2^ = 0.09, Power = 0.54, p = 0.040). This means that there was a decrease in retention in all three categories.

The MR group had a significant decline when comparing the mixed type questions score of anatomy test 1 and anatomy test 2 by 0.90 points (η_p_^2^ = 0.19, Power = 0.91, p = 0.002) (Fig. 2c). While there was no significant decline when comparing the anatomy test 1 to anatomy test 2 on the nominal type questions (0.32 points, η_p_^2^ = 0.02, Power = 0.17, p = 0.311) score and spatial type questions score (0.58 points, η_p_^2^ = 0.05, Power = 0.34, p = 0.119). This means that in nominal and spatial type questions the knowledge was retained but was not retained for the mixed type questions in the MR group.

### User preference, usability, and feedback

#### Questionnaire ratings

EFA results indicated that all items loaded significantly under each dimension with factor loadings of more than 0.30 (Table 1). Therefore, they were considered as reflective indicators of their respective dimensions. The Cronbach’s co-efficient alpha for user perception was quite high (α = 0.89). However, the internal consistency estimates for usability (α = 0.68) and ease of understanding (α = 0.63) were slightly lower than the commonly accepted threshold of 0.70. For the purpose of analyses reported in this study, these Cronbach co-efficient alpha estimates were still deemed acceptable since only three items were used for these factors.

**Table 1:** Exploratory factor analysis results and internal consistency estimates.

Dimension	Items	Factor loadings	Cronbach co-efficient alpha
**User perception**			0.89
	I found the learning session enjoyable.	0.94	—
	I found the learning tool to be exciting.	0.89	—
	I found the learning tool to be of high quality.	0.74	—
	I was engaged with the learning tool I received.	0.73	—
**Usability**			0.68
	I was able to focus on learning.	0.88	—
	I found the learning tool easy to use.	0.66	—
	I felt dizzy during the learning session.	0.42	—
**Ease of understanding**			0.63
	I found the text easy to understand.	0.90	—
	I found the images in the text easy to understand.	0.53	—
	I found the models in the learning tool easy to understand.	0.42	—

To examine the perception of each learning tool, Table 2 shows the average ratings by treatment group in various aspects of user perception, usability, confidence, long-term retention and memorability. The participants in the MR group had self-reported significantly higher ratings compared to the text-only group in the domains of user perception, long-term retention, and memorability. The 3DM group responses were significantly higher than the text-only group in the domains of user perception and usability.

**Table 2:** Descriptive statistics and ANOVA results.

	Text-only group		3DM group		MR group		Comparison		
	*M*	*SD*	*M*	*SD*	*M*	*SD*	*F*	*p*	Post-hoc analysis^a^
**User perception**	2.75	0.70	3.72	0.69	4.30	0.85	19.81	<0.001	MR > TO 3DM > TO
**Usability**	3.72	0.61	4.42	0.53	3.88	0.98	2.17	0.027	3DM > TO
Ease of understanding	3.00	1.11	3.38	0.80	3.51	0.86	1.44	0.248	—
Confidence									
I feel I did well on the test.	2.00	0.77	2.07	0.88	2.00	1.05	0.03	0.972	—
How difficult did you find this test? (5 = difficult, 1 = easy)	4.47	0.62	4.40	0.63	4.37	0.68	0.11	0.892	—
Long-term retention									
**How effective do you perceive the learning session was in helping you with long-term retention?** (5 = effective, 1 = not effective)	2.24	1.20	2.47	1.19	3.26	1.19	6.24	0.004	MR > TO
Memorability									
**How memorable was the learning session?** (5 = memorable, 1 = not memorable)	2.71	1.10	2.87	1.13	3.74	1.15	7.19	0.002	MR > TO

^a^Only includes comparisons with significant comparisons using Bonferroni post-hoc analysis.

Note: MR = MR group; 3DM = 3DM group; TO = Text-only group; specific domains have been underlined; significant comparisons are indicated in bold.

#### Observation and EEG evaluation

**Figure 3:**
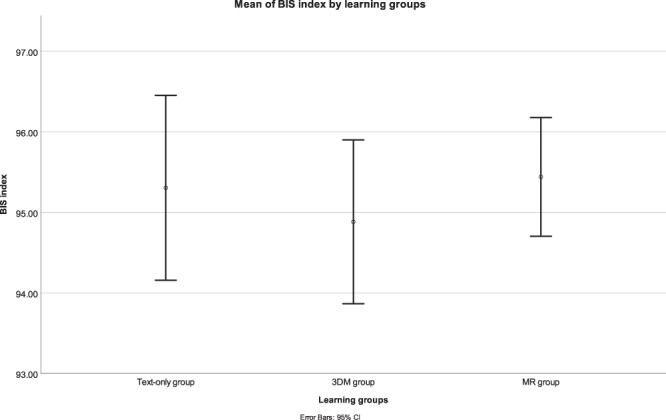
Mean BIS index by each learning group. Note: error bars represent the 95% confidence interval.

A one-way ANOVA for the BIS index showed no significant differences between the learning groups (*F*(2,49) = 0.374, *p* = 0.690). A summary of the means and standard deviations are shown in Fig. 3.

The MR group averaged a higher mean BIS index (*M* = 95.44, *SD* = 1.53) throughout the learning session compared to the 3DM group (*M* = 94.88, *SD* = 1.84) and the text-only group (*M* = 95.31, *SD* = 2.31). Overall, this suggests that MR as a learning tool is not inferior in its affordance of maintaining awakeness level compared to the text-only or 3DM learning style.

#### Qualitative feedback

Table 3 shows the main themes identified within each treatment group for each topic of interest when analyzing the open responses from the participants.

**Table 3:** Summary of themes identified from open responses by each treatment group about each topic of interest.

Topic of interest	Main themes identified		
	Text-only group	3DM group	MR group
Engagement	•Diagrams •Confusing •Good structure •Ability to write on the text	•Interesting •Lack of time	•Novel •Lack of time •Immersion
Excitement	•Boredom •Lack of novelty •Lack of visualization •Interesting content	•Clear visualization •Helpful animation •Familiarity •Difficult to use •Lack of interactivity	•Novelty •Lack of time •Helpful visualization and animation •Overwhelming
Long-term retention	•Lack of revision •Lack of interactivity •Lack of time	•Lack of revision •Helpful for spatial understanding •Unhelpful for nominal	•Lack of orientation •Helpful for spatial learning •Unhelpful for nominal learning •Distracted •Overwhelmed
Memorability	•Unhelpful for spatial or nominal retention •Lack of time	•Experience •Unable to remember content •Boring •Helpful for spatial retention •Engaging for ‘visual learners’	•Novel •Exciting •Helpful for spatial retention •Unhelpful for nominal retention
Exam difficulty	•Difficult •Lack of revision •2D images unhelpful for spatial knowledge	•Difficult •Visualization helpful for spatial knowledge	•Difficult •Visualization helpful for spatial knowledge •Lack of time •Focus more on spatial information

The text-only group participants felt that the text had a good structure on how the information was presented and enjoyed the ability to write onto the text. However, they became bored with this learning tool and some found a lack of interactivity from the text. Many of them had a difficult time with learning spatial knowledge and confusing to learn from.

Some participants using the 3DM device felt a sense of familiarity with this device and was interesting for them to use. However, not everyone agreed with some finding the application difficult to use and still lacked interactivity. Many participants felt that the 3D visualizations and animations were helpful in learning spatial information with some commenting that it was unhelpful for learning nominal information.

The participants in the MR group found the learning tool to be immersive which helped their engagement. They also found the device to be novel and exciting to use. They felt that the holographic visualizations and animations were helpful for learning spatial knowledge. However, the introduction of this novel tool meant they were unfamiliar with these tools and some found it overwhelming to use it as they had to learn the content and how to integrate the tool for their own learning. As well, for some participants, the novelty was so high that they became distracted by the holographic visualizations instead of learning and found that it was unhelpful for nominal learning.

When the participants in the text-only group and 3DM group were reflecting back on session 1 a month later, they felt that the lack of revision was the major issue contributing to the exam being difficult. However, this was not mentioned in the MR group which may indicate the students did not have enough familiarity with the tool and did not consider it normal to revise with this device.

## Discussion and Conclusion

To the best of our knowledge, this is the first study that has explored both short- and long-term (one month) learning outcomes based on textbook-style, 3DM, and MR learning tools. Further novel exploration includes the use of EEG data to quantitatively measure the awakeness of learners using the different modalities. The study was carried out with medical students who were randomly assigned to the three learning tools and tests were conducted immediately after the learning session and another a month later. The participants’ psychometric abilities, academic abilities, EEG data were all recorded to identify similarities and differences between the three groups. Overall significant differences were observed when comparing these learning modalities in terms of learning outcomes for anatomy teaching.

Short-term nominal type knowledge was only superior in text-only and 3DM group when compared to the MR cohort. A month later however this superiority disappeared, while on the other hand, the MR group was able to retain nominal and spatial type knowledge. The text-only group only managed to retain spatial type knowledge and the 3DM performed the worst in terms of retention where the group was unable to retain either type of knowledge. The short-term inferiority from the MR learning tool compared to the other groups was likely due to a lack of time and orientation with the MR device which was indicated through student open responses. Similarly, comparisons of the knowledge types and psychometric tests provided some interesting findings where specific psychometric abilities benefitted learning from different tools. Participants with better attention scores also did better in nominal type questions using the text-only learning tool. While, those with better spatial short-term memory performed better in spatial type questions when using the MR learning tool.

With regards to other factors that may have affected learning, the MR learning tool had the highest rated dizziness with the highest standard deviation, meaning it may cause the user dizziness, but this varies between users. Dizziness and other side effects of VR/AR tools also been reported in other experiments^13,32^. Additionally, MR and VR technologies have been shown to cause a vergence–accommodation conflict which limits performance and is strenuous^36^.

It was also shown that dizziness has a significant but weak relationship with nominal performance. This could explain some of the decreased performance of the MR participants compared to the text-only or 3DM group for anatomy test 1 nominal type questions. However, self-reported dizziness does not seem to correlate with the performance in the anatomy test after a month. Some further challenges with using the MR tools include physical discomfort on some participants as it was “quite heavy” on their heads and the requirement of orientation to the tool. It is also interesting that “lack of revision” was not the main problem for the 3D learning tool whereas it was the main problem identified in the textbook-style and 3DM learning tool. This could be because there were other more pressing problems such as “lack of orientation” with the tool or it was too “distracting”. Or it could have been of little concern as “it was still vivid” in their minds after a month.

Although the MR tool had mixed user effects, the participants found the MR visualization to be most engaging quantitatively comparing mean ratings of the learning tools with textbook-style and 3DM. The participants suggested a few reasons for their increased engagement such as greater immersion, clarity of the learning tool and the novelty of the technology which agrees with past literature^4,26,29–31^. This is also consistent with previous research on user acceptance of MR learning tools^26,29^. It was also interesting to see that despite the MR learning tool being rated more difficult to use than the 3DM learning tool, participants still found the learning tool to be of higher quality and more enjoyable than 3DM. This reinforces the power of novel technologies from past studies^37,38^. It is also noteworthy that the novelty can also negatively affect learning outcomes as they get too excited and fascinated by the technology they forget about learning from the tool. This was reinforced by a lower rated mean of the ability to focus when using the MR device compared to the 3DM learning tool.

The textbook-style learning, when compared with 3DM and MR learning tools, had consistently lower mean ratings in engagement, excitement, enjoyability, ability to focus on learning, ease of use, and quality of learning tool. This may be because many participants felt the learning session was “standard”, “boring” and cumbersome to read. Participants also noted that the 2D images were more difficult to understand than when there is access to 3DM and MR learning tools which is agreed by past studies^39,40^. However, without the adjuvant 3D visualizers, the text was rated to be easier to understand; possibly because the participants had more time to spend on the text during the learning session or they used the 3D visualizer learning tools as an anchor in responding to the question. The EEG BIS data also suggests that the use of the MR device is potentially superior to 3DM and text-only learning tools in the awakeness of the learner.

As a teacher or facilitator, it can be used to decrease errors in communication of spatial information, which is an affordance mentioned by past papers^41,42^ and enable students to consistently have access to the 3D spatial relationships. A suggestion for the implementation of 3D visualizers includes providing an orientation of the material and learning device. To begin, textbook-style learning tools should be provided to students to allow them to become familiar with the terminologies of the topic as done so by past research^43^. As well, when introducing the MR as an adjuvant for learning, it is important to provide an orientation to the new learning tool for the purpose of allowing students to understand how MR can integrate with their learning and reduce the novelty effect so they do not become distracted by fascination but are still excited to use the new learning tool^21^.

In conclusion, this study compared students’ learning experience when using various tools: textbook-style, 3DM and MR. The study employed qualitative and quantitative approaches to examine knowledge acquisition and long-term knowledge retention after a month. The results of this study suggest that while various emergent digital learning tools can support students’ learning experience in anatomy education, an enhanced learning experience is dependent on familiarization and orientation with these tools. More specifically, the results of this research add to the potential research in support of the potentials of utilising multidimensional tools in anatomy education. However, the study has a number of limitations.

## Limitations

The study sample size of 52 participants could be considered as a small sample size and thus should be interpreted with this in mind. A random number generator was utilized to randomly allocate participant groups which meant there would be no pattern in how they were assigned. However, this does not guarantee even groupings. As such, gender or other potential confounders which may influence spatial ability^44,45^ may not be matched.

Another limitation is the subject chosen and generalizability of the subject should be done with caution. It has already been identified that there are different types of anatomy knowledge (e.g. nominal and spatial). The specific topic chosen here was the visual neural pathway in anatomy which can be considered spatially complex. This should be kept in mind when generalizing to other areas of anatomy or subjects outside of anatomy as it may require a different balance in various types of knowledge.

It was also commented on by a few participants that the learning session was too pressured, especially for those using the MR learning tool. Some of the participants suggested it was because they were still understanding how to use the MR learning tool, or the holograms were too mesmerizing they did not have enough time in the learning session to study satisfactorily. The timing of one hour for the first session was because of the time limitation of the study and the need to go through participant individually for the first session. For the MR group, the learning session may be underestimated due to having less time to learn the material. While the text-only group and 3DM group were more likely to have rote learned instead of understanding the content thus leading to their decrease in performance from anatomy test 1 to anatomy test 2.

The question items of anatomy test 1 and anatomy test 2 were the same. However, to minimize the test effect we had randomized the order of the items in both tests; confirm with the students that no related study was complete between session 1 and session 2; and did not provide a learning session between anatomy test 1 and anatomy test 2. It should also be noted that a pre-learning session anatomy test was not undertaken for the purpose of minimizing the test effect, instead, their grade point average of anatomy papers was used to compare their baseline anatomical abilities.

All participants had self-reported not completing any study related to the learning session. Although this is self-reporting, some participants had also anecdotally stated there was no incentive from them to study for this test. The results were anonymized and thus did not count towards any summative grade and they would not be provided feedback from the tests.

Finally, eight psychometric tests were completed in one after the other in the second follow-up session with the participants. This could have also resulted in fatigue of the participants which lead to them not fully concentrate or complete the psychometric tests to the best of their abilities. To reduce the effect of fatigue, psychometric tests were completed in random order.

## Supplementary information

Supplementary information.
