# Supplementary material for: A Randomised Control Trial and Comparative Analysis of Multi-Dimensional Learning Tools in Anatomy
Source: Sci Rep. 2020 Apr 9;10:6120. doi: 10.1038/s41598-020-62855-6 (PMC7145854; doi:10.1038/s41598-020-62855-6)
Supplement: Supplementary file 1 — Supplementary information. [file 41598_2020_62855_MOESM1_ESM.pdf]

A Randomised Control Trial and Comparative Analysis of Multi-Dimensional Learning Tools in Anatomy

Authors List:

Chris Wang[1], Ben Kei Daniel[2,+], Mustafa Asil[3,+], Prashanna Khwaounjoo[1] and Yusuf Ozgur Cakmak[1,4,5,6]

Author Affiliations:

[1] Department of Anatomy, School of Biomedical Sciences, University of Otago, Dunedin, New Zealand
[2] Education Technology Group, Higher Education Development Centre, University of Otago, Dunedin, New Zealand
[3] Centre for Healthcare Education and Research Innovation (CHERI), School of Medicine, Medical Sciences and Nutrition, University of Aberdeen, Aberdeen, United Kingdom
[4] Brain Health Research Centre, Dunedin, New Zealand
[5] Medical Technologies Centre of Research Excellence, Auckland, New Zealand
[6] Centre for Health Systems and Technology, University of Otago, Dunedin, New Zealand
[+]these authors contributed equally to this work

Supplementary file for A Randomised Control Trial and Comparative Analysis of Multi-Dimensional Learning Tools in Anatomy

Appendix 1 – Demographics and academic abilities survey

Please enter your unique participant code:

01

Quick Question!

Would you like to be informed (via email) of the results at the end of the study?

Yes

No

02

Demographics

Are you:

Male

Female

Gender Diverse

Prefer not say

Which ethnicity do you identify as?

European

Māori

Pacific Peoples

Prefer not say

Asian

Middle Eastern/Latin American/African

Other:

Entry Pathway:

Undergraduate Pathway

Postgraduate Pathway

Alternative Pathway

Undergraduate Pathway:

General Pathway

Rural Pathway

Māori Pathway

Pacifica Pathway

Please enter your age:

What is your previous experience / exposure of knowledge in Anatomy?

Which class stream are you part of in ELM2?

A stream

B stream

C stream

D stream

03

Technology

Have you used the Microsoft HoloLens before?

Yes

No

Please indicate how much you would agree or disagree with this statement:

"I am very effective in using the Hololens."

Note effective				Effective
1	2	3	4	5

Please indicate how much you would agree or disagree with this statement:

"I learn new technologies easily"

Disagree				Agree
1	2	3	4	5

04

Academics

How do you perceive your **knowledge of anatomy**?

Low				High
1	2	3	4	5

How do you perceive your **general academic ability**?

Low				High
1	2	3	4	5

What was your HUBS**191** overall mark?

▼

What was your HUBS**192** overall mark?

Additional Comments:

Please feel free to add any additional comments.

Powered by Qualtrics

Appendix 2 – Anatomy test question items (Anatomy test 1)

Please write clearly your unique code: ____________________

1. If the optic chiasm was damaged, **draw** the likely **visual field defect**:

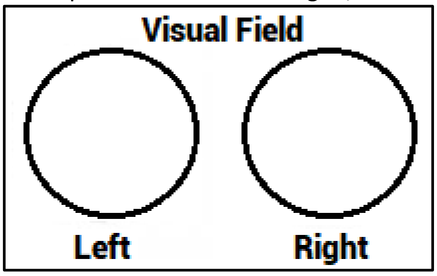

2. Which lobe of the brain contains the visual cortex?

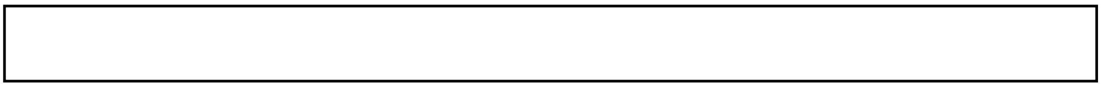

3. A tumor was discovered on the anterior aspect of the posterior horn of the ventricle. Please name the likely visual field defect.

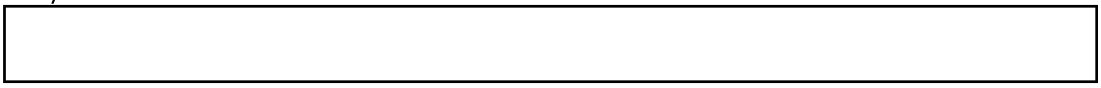

4. Please draw the visual field defect that results when there is damage to the left optic nerve.

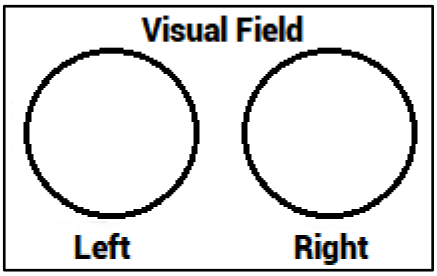

5. Please shade in which area of the **retina** which is involved in seeing an object in the top left quadrant of the visual field (as shown below):

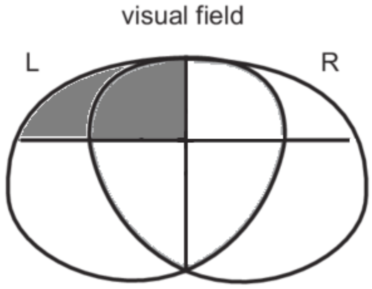

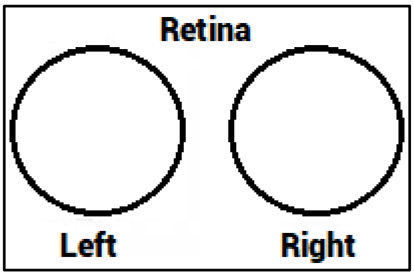

6. Where is the position of the pituitary gland from the perspective of the optic chiasm?

7. What is the name of the visual field defect that results when there is damage to the left optic nerve?

8. What is the name of the brain region superior to the calcarine sulcus?

9. What is the name of the brain region inferior to the calcarine sulcus?

10. Where is the position of the Meyer's loop from the perspective of the anterior horn of the lateral ventricle?

11. Which direction (relative to the lateral genicular nucleus) does the optic tract come into the lateral geniculate nucleus?

12. What is the name of the overall visual neural pathway structure between the lateral geniculate nucleus and the visual cortex?

13. What is the name of the visual neural pathway between the optic chiasm and the lateral geniculate nucleus?

14. Which quarter of the **retina** can perceive the shaded region of the visual field (shown below)?

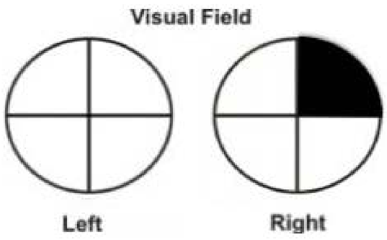

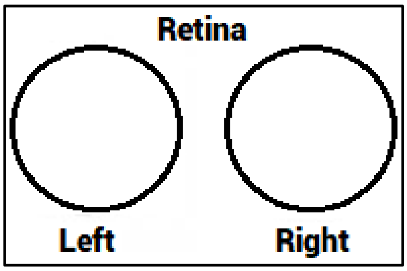

15. Describe the position of Meyer's loop relative to the posterior horn of the lateral ventricle.

16. Describe the position of Baum's loop relative to the posterior horn of the lateral ventricle.

17. If the right lateral geniculate body was damaged, **name** the likely **visual field defect**.

18. What is name of the visual neural pathway between the retina and optic chiasm?

Appendix 3 – Anatomy test marking rubric

Supplementary Table S1. Anatomy test marking rubric. Note: Only correct answers were awarded a point. Answers which were partially correct, incorrect, or empty were awarded no points.

Matrix type	Question ID	Question	Correct [C]	Partially Correct [S]	Incorrect [X]	Empty [E]
Nominal, Easy	1	Which lobe of the brain contains the visual cortex?	Occipital lobe	Posterior lobe of brain	-	Only includes: [empty spacec], or “?”, or “-”
Nominal, Easy	2	What is name of the visual neural pathway between the retina and optic chiasm?	Optic nerve	Any of the correct words	-	-
Nominal, Medium	3	What is the name of the visual neural pathway between the optic chiasm and the lateral geniculate nucleus?	Optic tract	Any of the correct words	-	-
Nominal, Medium	4	What is the name of the overall visual neural pathway structure between the lateral geniculate nucleus and the visual cortex?	Any of:<br>• Optic radiation<br>• Meyer's loop<br>• Baum's loop	Any of the correct words	-	-
Nominal, Difficult	5	What is the name of the brain region superior to the calcarine sulcus?	Cuneus (gyrus)	Occipital lobe	-	-
Nominal, Difficult	6	What is the name of the brain region inferior to the calcarine sulcus?	Lingual (gyrus)	Occipital lobe	-	-

Mixed, Easy	7	Please draw the visual field defect that results when there is damage to the left optic nerve.	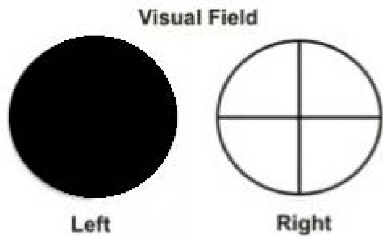	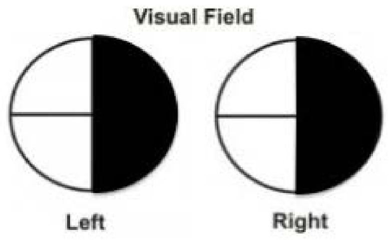	-	-
Mixed, Easy	8	What is the name of the visual field defect that results when there is damage to the left optic nerve?	Any of:<br>• Blindness in left eye<br>• Left monocular blindness	Any of:<br>• Can't see / Blind	-	-
Mixed, Medium	9	If the right lateral geniculate body was damaged, **name** the likely **visual field defect**.	Implies (also accept contralateral in place of left and left bilateral) including all of:<br>Left Homonymous Hemianopia	Implies (also accept bilateral):<br><br>Homonymous Hemianopia	-	-
Mixed, Medium	10	If the optic chiasm was damaged, **draw** the likely **visual field defect**:	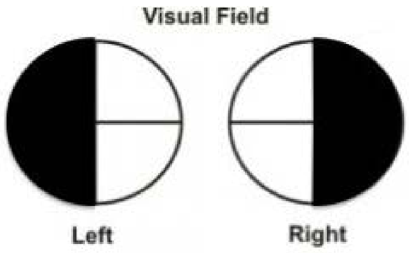	Any of:<br>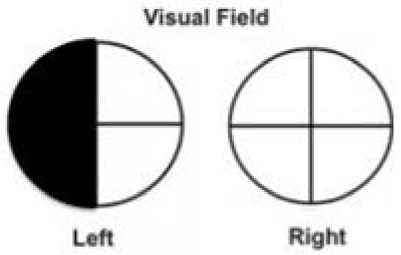<br>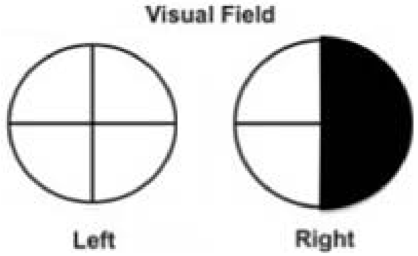	-	-
Mixed, Difficult	11	A tumor was discovered on the anterior aspect of the posterior horn of the	All of:<br>Homonymous inferior quadrantanopia	Implies (e.g. accept bilateral):<br>Homonymous quadrantanopia	-	-

		ventricle. Please name the likely visual field defect.				
Mixed, Difficult	12	▪ Please shade in which area of the **retina** which is involved in seeing an object in the top left quadrant of the visual field (as shown below):<br>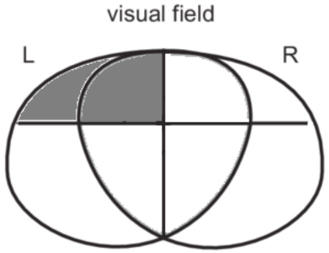	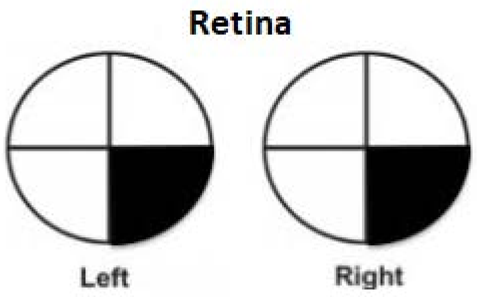	Any of the below:<br>• 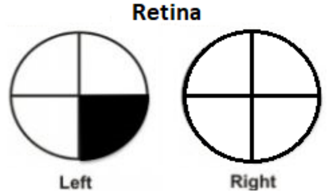<br>• 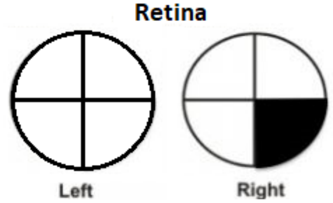	-	-
Spatial, Easy	13	Where is the position of the pituitary gland from the perspective of the optic chiasm?	Includes/implies:<br>• Pituitary is inferior to optic chiasm<br>• Also accept: postero-inferior	Implies:<br>• Pituitary gland is posterior	-	-
Spatial, Easy	14	Which direction (relative to the lateral geniculate nucleus) does the optic tract come into the lateral geniculate nucleus?	Implies (and without contradictory terminology to any of the following words):<br>• Anteriorly or anterolaterally<br>• Ventrally or ventrolaterally	Implies:<br>• Lateral	-	-
Spatial, Medium	15	Where is the position of the Meyer's loop from the perspective of the **anterior** horn of the lateral ventricle?	At least two of the following (and without contradictory terminology to any of the following words):<br>• Posterior<br>• Lateral<br>• Inferior	Only one of the following (and without contradictory terminology to any of the following words):<br>• Posterior<br>• Lateral<br>• Inferior	-	-

Spatial, Medium	16	Which quarter of the **retina** can perceive the shaded region of the visual field (shown below)?<br>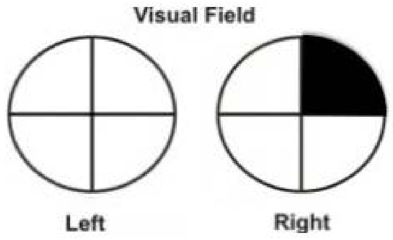	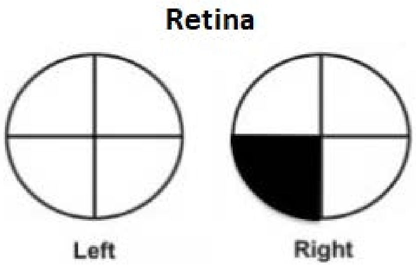	Any of:<br>• 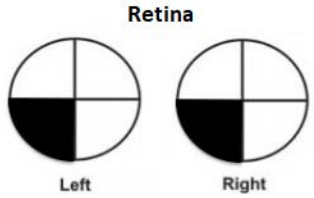<br>• 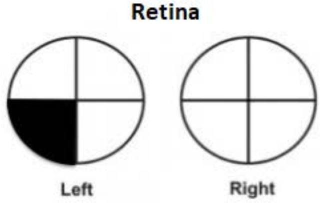	-	-
Spatial, Difficult	17	Describe the position of Baum's loop relative to the posterior horn of the lateral ventricle.	All of the below (and without contradictory terminology to any of the following words; e.g. anterior and posterior are not contradictory):<br>• Lateral<br>• Superior	Only one of the below (and without contradictory terminology to any of the following words; e.g. anterior and posterior are not contradictory):<br>• Lateral<br>• Superior	-	-
Spatial, Difficult	18	Describe the position of Meyer's loop relative to the posterior horn of the lateral ventricle.	All of the below (and without contradictory terminology to any of the following words; e.g. anterior and posterior are not contradictory):<br>• Lateral<br>• Inferior	Only one of the below (and without contradictory terminology to any of the following words; e.g. anterior and posterior are not contradictory):<br>• Lateral<br>• Inferior	-	-

Appendix 4 – User perception and usability survey

Please enter your unique participant code:

Which Learning Session did you receive?

01

How was your experience?

How much do you agree with the following statement: "**I was engaged with the learning tool I received.**"

Disagree				Agree
1	2	3	4	5

Please briefly explain your choice to the question above:

How much do you agree with the following statement: "**I found the learning tool to be exciting.**"

Disagree				Agree
1	2	3	4	5

Please briefly explain your choice to the question above:

02

Please indicate to what extent you agree with the following statements:

"I found the learning tool easy to use."

Disagree				Agree
1	2	3	4	5

"I found the learning tool to be of high quality."

Disagree				Agree
1	2	3	4	5

"I felt dizzy during the learning session."

Disagree				Agree
1	2	3	4	5

"I found the learning session enjoyable."

Disagree				Agree
1	2	3	4	5

"I was able to focus on learning."

Disagree				Agree
1	2	3	4	5

"I found the **models** in the learning tool easy to understand."

Disagree				Agree
1	2	3	4	5

"I found the **images** in the text easy to understand."

Disagree				Agree
1	2	3	4	5

"I found the text easy to understand."

Disagree				Agree
1	2	3	4	5

"I feel I did well on the test."

Disagree				Agree
1	2	3	4	5

Additional Comments:

Powered by Qualtrics

Appendix 5 – Memorability and long-term retention survey

Please enter your unique participant code:

01

Final Survey

Have you done any study related to the test?

Yes

Maybe

No

Please indicate what you had studied and how much you studied:

02

Last few questions

How difficult did you find this test?

Easy				Difficult
1	2	3	4	5

Please explain your choice above:

How memorable was the learning session?

Not memorable				Memorable
1	2	3	4	5

Please explain your choice above:

How effective do you perceive the learning session was in helping you with long-term retention?

Not effective				Effective
1	2	3	4	5

Please explain your choice above:

Additional Comments:

Please feel free to add any additional comments.

Powered by Qualtrics

Appendix 6 – Identified themes and example quotations

Supplementary Table S2. Themes and example quotations by learning group.

Theme	Learning group	Examples of quotations
Engagement	Text-only	"It was quite difficult to read through sentences with many words that I hadn't even heard of and trying to construct a picture in my mind or figure out from the diagram what it was describing"<br>"Had all the information in one handy place to learn, but reading off a page is boring and not engaging. Limited interactions and hard to learn anatomy when you can't see and manipulate the structures."<br>"Personally enjoy being able to write on the material as I study"
	3DM group	"The images on the laptop were set out in a way that made the information that I had read easy to translate into a physical understanding of the structures, and the pathways. The laptop images were the main engaging aspect."<br>"It was good seeing the anatomy in 3D and being able to see where light information would go an how it be processed in the brain. When looking at the model I understood it, I am not sure how much of it I was able to retain though. It would just require more time."
	MR group	"Holographic was a unique experience to learn the neural pathway of the brain. I felt engaged and it was nice to be able to stand up and walk around the models."<br>"-Very shakey visual field within the hologram<br>-Not enough time to look at the holograms<br>-Unsure what I was looking at with the holograms and what to focus on<br>-Not clear what certain things in the hologram meant - e.g. the moving balls"
Excitement	Text-only	"I felt that paper was limiting in the way that there was a limited number of diagrams with specific perspectives and wasn't particularly suitable for learning anatomy - I tend to use a textbook with anatomy TV on my laptop so that I can see how the things I read in the textbook fit together in 3D (which is usually more complex than words can describe e.g. not quite posterolateral etc.)"<br>"Just standard textbook info - the topic is interesting though."<br>"The textbook material was less exciting and less stimulating as compared to a 3D model or a hologram. As it is quite a typical resource, it did not spark much interest in me. That might have affected by ability to grasp the information. I found my attention wandering off after a few minutes of reading the textbook material."

Theme	Learning group	Examples of quotations
Excitement	3DM group	"It complemented the text quite well, it was much easier to visualize using the problems using the software"<br>"I liked the animation aspects as I haven't seen those in 3D models before (for anatomy at least), even though the 3D models are fairly common. Still, it's always great to see how technology progresses."<br>"It was fun to move the mouse around but I am not a gamer nor do I use computers for more than University notes. It felt like I was playing a computer game. The mouse was hard to control and manouevre. The tool had no interactive components (ie. no games, quiz or animations). You were only changing the view. It was a new way of learning but I didn't appreciate it. I don't think it was a very effective use of my time studying via this method."
	MR group	"It's new, it's exciting. Need more time to learn how to use it for it to be more useful than computer study"<br>"I found it more stimulating being able to see structures and pathologies we wouldn't be able to see with gross structures in labs (wets, etc.)."<br>"Compared to VR augmented reality wasn't engaging. Overloaded with new terms and also trying to use a new learning tool. Didn't enjoy this method of learning"
Long-term retention	Text-only	"Maybe could have done better with more time - so that I had the time to think through what I was reading and visualise it"<br>"I didn't recall much long term from reading some text for a short time a while ago, and not looking back over it again."<br>"I learn in a more interactive manner which is not really provided by textbooks."
	3DM group	"Very useful in terms of understanding what is occurring and giving a deeper knowledge behind it but it is hard to memorize the names like geniculate nucleus without repeated exposure to it which is done usually by more conventional means."<br>"Short time frame made it limited, however the information was clear"<br>"More helpful than 2d images are you are able to comprehend the diagrams in space a lot better"

Theme	Learning group	Examples of quotations
Long-term retention	MR group	“I feel like the session has a lot of potential to be effective. I didn't go about the first session in the right way because I think I should have used the written information alongside the holograms, but I first read the information and then looked at the holograms. The learning session wasn't useful for remembering the names of different pathologies or specific parts of the visual pathways, but was good for remembering what parts of the retina are used for the different parts of the visual fields and having a spatial awareness of the structures in relation to each other.”<br>“Was pretty distracted by Holograms so I focused less on the actual learning. But once I got used to it, I think it would be more effective.”<br>“Good for pathways, but more time would have been needed to learn names, but with a few more sessions I would be confident in understanding the mechanisms behind pathology.”
Memorability	Text-only	“I vaguely remember how visual fields crossed over but couldn't accurately recall this, nor did I remember the names of area's of the brain or where things are orientated in relation to each other.”<br>“I needed more time to absorb the information”<br>“I remembered it was hard and complex and there were some difficult names but otherwise but otherwise didn't really take in much...”
	3DM group	“I remember it was boring. I regretted participating in the study because I wasn't in the hologram group. I found the first session a complete waste of time.”<br>“I was surprised by how much I was still able to remember from the first session. I still had an image of the model in my head, but it was a bit blurred. And I still remembered a bit about the relative positions of things.”<br>“Really interesting, Given I am a visual learner, it was good to be able to interact with the visual fields.”
	MR group	“Three D image makes it more alive and vivid and approachable.”<br>“It was a very unique experience. I didn't retain much in terms of names but I could still remember what the hologram roughly looked like when coming to the second session and had a general idea of the pathways.”<br>“Mostly I was excited about using the hologram so I didn’t focus much on the academic part”
Exam difficulty	Text-only	“I did no study for this as I felt otherwise this would lead to an inaccurate representation of how my memory and learning was influenced by the choice of learning tool. It made the test obviously very hard as I had learnt the content a long time ago with no further revisiting.”<br>“Was very very difficult - especially the names for the pathologies and the relative positions of structures.”

Theme	Learning group	Examples of quotations
Exam difficulty	3DM group	"I had a hard time remembering some of the specific names, but I remembered being in the session and looking at them - so I could visualise the 3D program that I used, but I just couldn't place a name onto the structure that I was being asked about."<br>"Having only just learnt the content for the first time prior to the test made it difficult."
	MR group	"Difficult to retain such knowledge learnt in a short period over time."<br>"I have never learned anything about visual pathway before. when I was using the hologram I was more focus on the shape, structure, and color of the model but wasn't focusing on the name of each structure. therefore I had a hard time remembering the names of the anatomical structures."<br>"Had no knowledge of the content before doing the test. Found the content quite difficult - don't think it was related so much to the format but would have been nice to have understood what I was doing."

Appendix 7 – One-way ANOVA of baseline anatomy knowledge characteristics of each learning group

Supplementary Table S3. Comparing the baseline anatomy knowledge characteristics of each learning group. Human body systems are papers which all participants should have undertaken at the University of Otago before entering medical school. Note: the grade point average (GPA) follows the University of Otago GPA scale.

	Text-only group		3DM group		MR group		Comparison	
	M	*SD*	*M*	*SD*	*M*	*SD*	*F*(2, 46)	*P*
Human body systems 1 GPA mean	8.44	1.03	8.58	0.79	8.32	1.06	0.271	0.764
Human body systems 2 GPA mean	8.44	1.09	8.75	0.45	8.47	0.90	0.490	0.616
